# Does Provincial Pooling of the Resident Medical Insurance Fund Improve the Efficiency of Medical Resource Allocation of China?

**DOI:** 10.3390/healthcare14152368

**Published:** 2026-08-03

**Authors:** Fukun Zhu, Haoran Li, Austen El-Osta, Bin Zhu, Ying Mao

**Affiliations:** 1 School of Public Policy and Administration, Xi’an Jiaotong University, Xi’an 710049, China; zhu2003@stu.xjtu.edu.cn (F.Z.); h.li2@imperial.ac.uk (H.L.); 2 School of Public Health, Imperial College London, London W12 0BZ, UK; a.el-osta@imperial.ac.uk; 3 School of Public Health and Emergency Management, Southern University of Science and Technology, Shenzhen 518055, China; zhub6@sustech.edu.cn

**Keywords:** health insurance pooling, medical resource allocation efficiency, difference-in-differences, health system reform, China

## Abstract

**Background:** Provincial pooling of the Urban–Rural Resident Basic Medical Insurance (URRBMI) fund has been promoted in China to reduce fragmentation and improve system performance, yet its impact on the efficiency of medical resource allocation remains unclear. **Objective:** To evaluate the impact of provincial pooling on medical resource allocation efficiency and to examine its heterogeneity across regions and institutional arrangements. **Methods:** Using provincial panel data from 2010 to 2022, this study measures efficiency with a super-efficiency slack-based measure (SBM) model and applies a staggered difference-in-differences (DID) design to estimate policy effects. **Results:** Provincial pooling is associated with a statistically significant reduction in medical resource allocation efficiency among adopting provinces. In baseline specifications, the estimated effect ranges from −0.021 to −0.024 (*p* < 0.01), corresponding to a decline of 0.021–0.024 points in the efficiency score, equivalent to about 2.4–2.7% of the sample mean efficiency level. Heterogeneity analysis suggests that the negative effect is concentrated in western provinces, whereas no statistically significant effect is observed in eastern provinces. No treatment effect is estimated for central provinces because none implemented provincial pooling during the study period. Institutional heterogeneity analysis shows negative estimates across pooling and management models, but the revenue–expenditure pooling estimate is not statistically significant. These results are robust across alternative specifications and sensitivity analyses. **Conclusions:** These findings suggest that, among adopting provinces, provincial pooling may involve substantial transitional adjustment costs, implying that efficiency gains should not be expected automatically in the short to medium term. Instead, outcomes depend on institutional conditions and the alignment of complementary governance mechanisms. Policymakers should adopt regionally differentiated and phased strategies and strengthen incentive-compatible design to improve reform effectiveness.

## 1. Introduction

As healthcare systems worldwide continue to reform, improving the efficiency of medical resource allocation while promoting equity and financial sustainability has become a central concern in health policy [1]. Within health insurance systems, the level of risk pooling represents a fundamental dimension of institutional design, determining how financial risks are distributed across populations and administrative jurisdictions. In China, the basic medical insurance system (MIS) plays a fundamental role in the social security framework, with its operational efficiency closely linked to healthcare system performance and governance capacity [2]. However, the system has long been characterized by substantial regional fragmentation. The Urban–Rural Resident Basic Medical Insurance (URRBMI) fund has historically been managed at the county or municipal level, leading to significant disparities in financing capacity, benefit levels, and resource allocation efficiency. Variations in regional economic development and healthcare capacity further exacerbate inefficiencies and constrain the optimal allocation of medical resources [3,4].

To address these challenges, China has progressively advanced provincial pooling of the URRBMI fund since 2016 as part of a broader national strategy to improve the integration and governance of the health insurance system. The reform represents a continuation of China’s gradual transition from fragmented county- and municipal-level pooling toward higher-level integration of insurance funds and governance responsibilities. As part of this institutional transition, key functions—such as revenue collection, benefit design, and budget management—have been progressively shifted to the provincial level to expand risk pooling, strengthen strategic purchasing, and promote more equitable and efficient resource allocation. Evaluating whether provincial pooling achieves these intended objectives is therefore of substantial policy importance.

Existing literature has largely emphasized the benefits of increasing pooling levels. Risk pooling is widely recognized as a fundamental mechanism for health insurance financing, as larger pooling arrangements can improve risk diversification, enhance financial resilience, and strengthen the capacity of insurance systems to respond to unexpected health shocks [5]. The theoretical rationale is closely related to the “law of large numbers,” whereby expanding the size of the risk pool reduces expenditure uncertainty and improves mutual support among insured populations [6,7,8]. Existing research further suggests that integrated pooling arrangements can reduce fragmentation, promote equity in health financing, and improve the sustainability of health insurance systems [9]. In the context of China, previous studies have shown that raising the pooling level of basic medical insurance may reduce regional disparities, improve benefit equalization, alleviate medical cost burdens, and enhance healthcare access among insured populations. Empirical evidence from China further suggests that provincial pooling can reduce medical expenditure burdens and improve health outcomes by strengthening risk sharing and facilitating access to healthcare services [10,11,12,13,14]. However, increasing pooling levels may also generate unintended consequences by reshaping existing governance structures and incentive arrangements. The transfer of fund management responsibilities from lower administrative levels to higher-level pooling authorities may weaken local governments’ incentives for expenditure control and supervision, thereby increasing the risk of moral hazard [15,16,17,18]. Moreover, reduced monitoring capacity and misaligned incentives between insurance agencies and healthcare providers may contribute to excessive healthcare utilization, supplier-induced demand, and rapid expenditure growth, potentially threatening fund sustainability [19,20,21,22].

With respect to medical resource allocation efficiency, prior studies have primarily relied on technical efficiency approaches, such as Data Envelopment Analysis, to examine regional disparities and performance differences [23,24]. While overall efficiency has improved in China, significant regional inequalities persist, with higher efficiency in eastern regions and lower levels in western areas [25,26,27]. However, limited attention has been paid to how institutional reforms—particularly changes in pooling levels—affect efficiency outcomes.

Against this backdrop, important gaps remain. Existing studies focus predominantly on fund sustainability, equity, or risk-sharing capacity, while providing limited evidence on whether provincial pooling improves resource allocation efficiency. Moreover, substantial regional differences in economic structure, healthcare capacity, and institutional arrangements suggest that policy effects may be heterogeneous.

To address these gaps, this study examines the impact of provincial pooling of URRBMI fund on medical resource allocation efficiency in China. Specifically, this study addresses two objectives: (i) quantify the average effect of provincial pooling on medical resource allocation efficiency using a staggered difference-in-differences (DID) design; and (ii) examine whether the policy effects exhibit heterogeneity across different regional contexts and institutional arrangements, including regional variations, fund pooling models, and administrative management models.

## 2. Materials and Methods

### 2.1. Study Setting

This study focuses on the reform toward provincial pooling of the URRBMI fund in China. As a foundational medical security program covering more than one billion enrollees, the URRBMI has long been administered primarily at the county or municipal level. This relatively low pooling level has resulted in fragmented financing capacity and pronounced regional disparities in benefit levels and protection capacity. Constrained by local fiscal capacity and institutional foundations, different pooling units have exhibited persistent imbalances in risk-sharing capacity, purchasing power, and policy implementation effectiveness.

Initiated within a broader national health insurance governance reform agenda, the overarching reform objective is to gradually recentralize key functions—previously dispersed across county and municipal levels—such as fund revenue collection, benefit policy design, budget management, inter-regional fund adjustment, and payment arrangements, at the provincial level. Compared with the Urban Employee Basic Medical Insurance scheme, which operates at a higher pooling level and exhibits more mature institutional arrangements, the URRBMI is characterized by greater institutional heterogeneity and a higher reliance on government subsidies. As a result, it is more sensitive to changes in pooling arrangements during the reform process.

In terms of institutional design, provincial pooling varies along two dimensions. Fund arrangements include revenue–expenditure pooling, in which revenues and expenditures are managed provincially, and risk-adjustment pooling, in which local funds remain operational but participate in provincial redistribution. Administrative arrangements include vertical management under provincial authority and tiered management across provincial, municipal, and county levels (see Table 1).

The implementation of provincial pooling across China exhibits a typical “staggered adoption” pattern. Although the reform was promoted nationwide, provinces completed the transition at different points in time due to differences in administrative preparation, institutional foundations, and the complexity of integrating previously fragmented insurance management systems. Importantly, the timing of implementation was not determined by short-term changes in medical resource allocation efficiency. Instead, provincial adoption mainly reflected the gradual advancement of national reform requirements and differences in institutional readiness. The staggered implementation of provincial pooling provides an appropriate policy setting for conducting a staggered DID analysis, enabling this study to estimate the effect of provincial pooling on the efficiency of medical resource allocation within the policy implementation context.

### 2.2. Study Design

This study employs a multi-method empirical strategy to evaluate the impact of provincial pooling of the URRBMI fund on the efficiency of medical resource allocation across Chinese provinces from 2010 to 2022. First, following previous studies on healthcare resource allocation efficiency [28,29], we constructed a provincial-level panel dataset and employed a super-efficiency Slack-Based Measure (SBM) model to measure the annual efficiency of medical resource allocation. Second, based on variations in the timing of provincial pooling across provinces, we constructed a time-varying DID model to identify the causal effect of the reform. Finally, we conducted a series of robustness checks to ensure the reliability of the results.

### 2.3. Variables and Measurements

The dependent variable in this study is the efficiency of medical resource allocation, which is measured using a super-efficiency SBM model based on an input–output indicator system. Following previous studies on healthcare resource allocation efficiency [23,30], this indicator system evaluates how effectively healthcare resources are transformed into healthcare services at the provincial level. Therefore, the concept of efficiency examined in this study primarily reflects the technical efficiency of healthcare resource utilization, rather than overall healthcare quality or population health outcomes.

The indicator system incorporates multiple dimensions of healthcare resource inputs and service outputs to evaluate the efficiency of medical resource allocation across provinces. Healthcare resources mainly consist of human resources, physical infrastructure, and service capacity. Accordingly, this study incorporates health technicians, hospital beds, and medical institutions as input indicators, representing healthcare workforce, fixed infrastructure, and healthcare supply capacity, respectively [31,32]. For output indicators, healthcare services generated through these resources were measured using indicators reflecting service provision. Specifically, medical visits, inpatient services, and surgical procedures were included as desirable outputs, while mortality-related indicators were incorporated as undesirable outputs to capture differences in healthcare service outcomes [33]. Table 2 summarizes the input, desirable-output, and undesirable-output indicators used to construct the efficiency score.

The key independent variable in this study is the interaction term between the URRBMI provincial pooling policy indicator and the year of its implementation. The control variables include per capita GDP, social consumption level, the share of tertiary industry value added, population density, and the number of URRBMI enrollees, in order to account for differences in regional socio-economic conditions and insurance enrollment characteristics.

### 2.4. Data Source

The dataset was compiled from multiple authoritative sources, including the China Health Statistical Yearbook, China Statistical Yearbook, provincial statistical yearbooks, and policy documents issued by the National Healthcare Security Administration (NHSA) and provincial governments. Data on healthcare resource inputs, outputs, and provincial socioeconomic characteristics were extracted and matched at the province-year level. To ensure consistency across sources, variables were harmonized according to unified statistical definitions and reporting standards. The completeness of all analytical variables was carefully checked at the province-year level, and no missing-value imputation or interpolation was applied to the original balanced panel.

After data cleaning, harmonization, and validation, a balanced panel dataset covering 31 provinces over the period 2010–2022 was constructed, resulting in 403 province-year observations. This balanced panel served as the basis for efficiency measurement and subsequent policy evaluation analyses.

### 2.5. Statistical Analysis

To accurately assess inter-provincial differences in resource allocation efficiency, this study adopts the super-efficiency SBM model, which can effectively handle slack in input and output variables and distinguish frontiers beyond the efficiency boundary [34]. Compared with traditional DEA models, the SBM model provides more precise efficiency estimates by incorporating non-radial and non-oriented characteristics into the evaluation process [35]. The calculation formula is as follows:

(1) $$p=\min\frac{1-\frac{1}{m}\sum_{i=1}^{m}\frac{s^{-}}{x_{i_{0}}}}{1+\frac{1}{s_{1}+s_{2}}\left(\sum_{r=1}^{s_{1}}\frac{s_{r}^{g}}{y_{r_{0}}^{g}}+\sum_{r=1}^{s_{2}}\frac{s_{r}^{b}}{y_{r_{0}}^{b}}\right)}$$

(2) $$s.t.\left\{\begin{array}{l} x_{0}=X\lambda +s^{-},y_{0}^{g}\lambda -s^{g},y_{0}^{b}=Y^{b}\lambda +s^{b}\\ s^{-}\geq 0,s^{g}\geq 0,s^{b}\geq 0,\lambda \geq 0 \end{array}\right.$$

In the model, p represents the efficiency of medical resource allocation; m, g and b denote the numbers of input, desirable output, and undesirable output indicators, respectively; x, $y^{g}$ and $y^{b}$ represent the values of inputs, desirable outputs, and undesirable outputs; λ is the weight vector for inputs and outputs; and $s^{-}$, $s^{g}$ and $s^{b}$ are the slack variables for inputs, desirable outputs, and undesirable outputs. The efficiency measure p is monotonically decreasing in $s^{-}$, $s^{g}$ and $s^{b}$, with 0≤p≤1. A decision-making unit is considered efficient if and only if p=1.

On this basis, we apply a staggered DID design to estimate the policy impact. The approach leverages cross-provincial variation in the timing of URRBMI provincial pooling, treating provinces that adopted the reform earlier as the treatment group and those adopting it later or not at all as the control group. The empirical model is specified as follows:

(3) $$Efficiency_{it}=\alpha +\beta DID_{it}+\gamma X_{it}+\mu_{i}+\lambda_{t}+\varepsilon_{it}$$

In the model, $Efficiency_{it}$ denotes the efficiency of medical resource allocation in province i in year t. $DID_{it}$ represents the treatment indicator in the staggered DID framework, which equals 1 if province i has implemented provincial pooling of the URRBMI fund in year t, and 0 otherwise. Accordingly, the coefficient β captures the average treatment effect of provincial pooling on medical resource allocation efficiency. $X_{it}$ is a vector of time-varying provincial control variables, including per capita GDP, social consumption level, the share of tertiary industry value added, population density, and the number of URRBMI enrollees. α represents the intercepted item. $\mu_{i}$ captures province fixed effects, controlling for time-invariant provincial characteristics, while $\lambda_{t}$ captures year fixed effects, accounting for common time trends. $\varepsilon_{it}$ is the random error term. The efficiency scores were calculated using R version 4.3.2. All subsequent statistical analyses were performed using Stata 18.0.

## 3. Results

### 3.1. Descriptive Statistics

Table 3 presents the descriptive statistics of the indicators used in the SBM model. The results show substantial variation in healthcare resource inputs and service outputs across provinces. Healthcare inputs, including medical institutions, health technicians, and hospital beds, exhibit considerable regional disparities, while service outputs and quality-related indicators also vary markedly across provinces. Overall, the descriptive statistics suggest significant heterogeneity in healthcare resource allocation and service provision across China, providing a foundation for subsequent efficiency analysis.

Table 4 presents the descriptive statistics of the variables used in the staggered DID analysis. The mean efficiency score is 0.890, suggesting that the overall efficiency of medical resource allocation remained relatively high during the study period. Considerable variation is observed across provinces in terms of economic development, industrial structure, population density, and insurance enrollment.

### 3.2. Efficiency of Medical Resource Allocation in Each Province

Medical resource allocation efficiency across 31 provinces from 2010 to 2022 exhibited substantial heterogeneity, with clear phase-specific patterns over time. Most provinces maintained efficiency scores between 0.80 and 0.95, while a small number approached or reached the efficiency frontier (efficiency = 1). Around 2020, several provinces experienced a pronounced decline, followed by a subsequent rebound. These temporal fluctuations coincided with the COVID-19 pandemic period, although the descriptive trends alone do not allow causal attribution to the pandemic.

**Figure 1:**
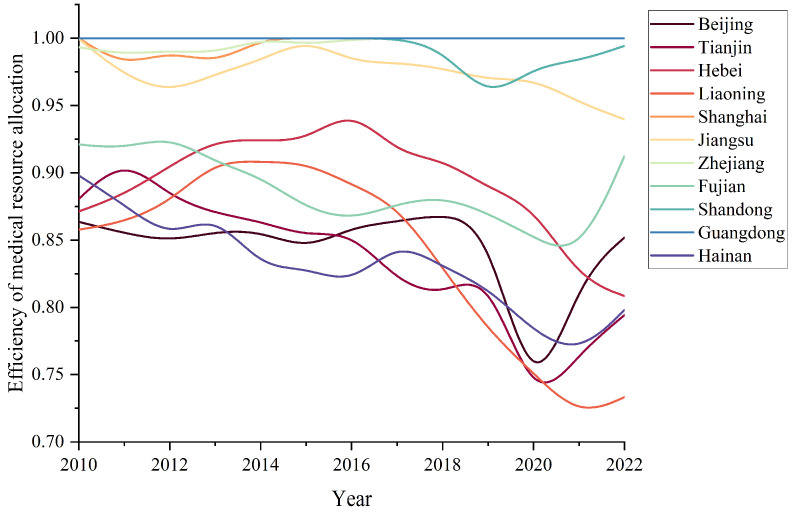
Medical resource allocation efficiency trends in eastern provinces, 2010–2022.

Figure 1 presents the efficiency trends for the 11 eastern provinces. Overall, the eastern region exhibited relatively high efficiency levels, with most provinces maintaining scores above 0.85 over the long term. Provinces such as Beijing, Shanghai, and Guangdong remained close to the efficiency frontier with minimal fluctuations, indicating relatively stable medical resource allocation efficiency in the eastern region over the study period.

**Figure 2:**
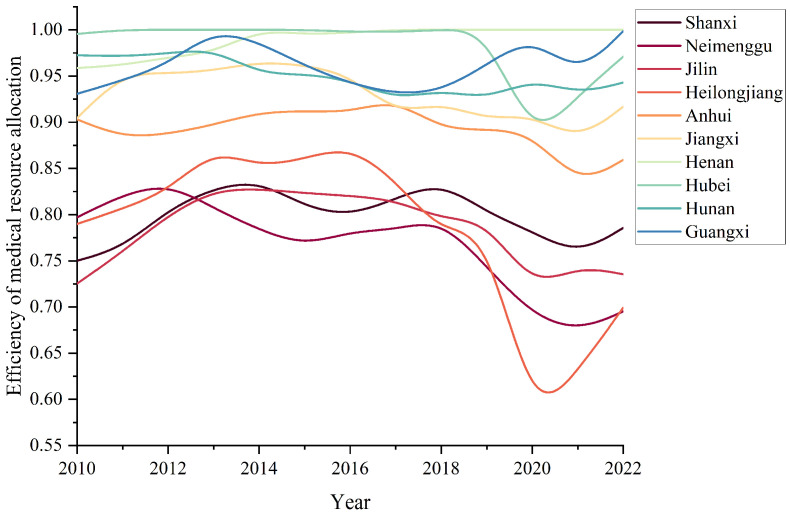
Medical resource allocation efficiency trends in central provinces, 2010–2022.

Figure 2 displays the efficiency trends for the 10 central provinces. The central region generally shows moderate efficiency levels compared with other regions, with most provinces fluctuating within the range of 0.75–0.90.

**Figure 3:**
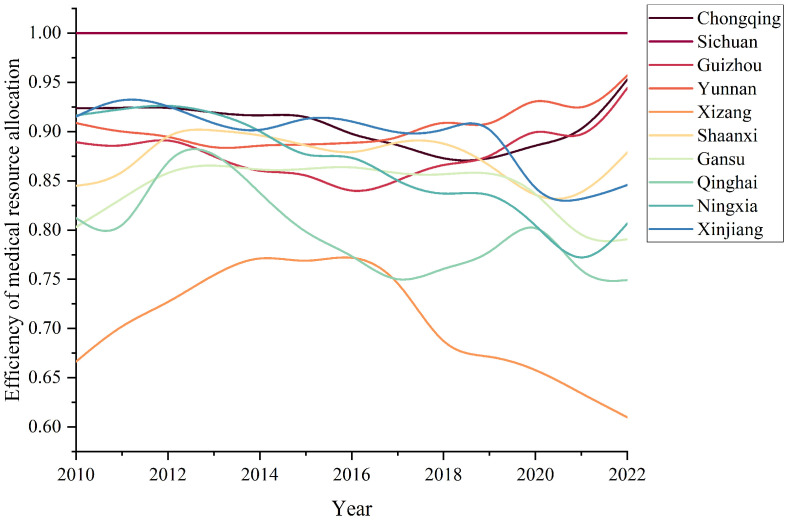
Medical resource allocation efficiency trends in western provinces, 2010–2022.

Figure 3 illustrates the efficiency trends for the 10 western provinces. Compared with the eastern and central regions, the western region exhibits overall lower efficiency levels, with most provinces falling within the range of 0.70–0.90, and several provinces remaining at relatively low efficiency levels for an extended period.

### 3.3. Staggered Difference in Difference Regression Results

Table 5 reports the baseline regression results on the impact of URRBMI provincial pooling on the efficiency of medical resource allocation.

The DID estimates show a statistically significant negative association between the provincial pooling reform of the URRBMI and medical resource allocation efficiency. Without controlling for additional covariates, the DID coefficient is –0.021 and statistically significant at the 1% level, suggesting that the reform was associated with an average reduction of 0.021 points in the efficiency score. After incorporating controls for per capita GDP, social consumption level, industrial structure, population density, and insurance enrollment, the absolute value of the DID coefficient increases slightly to −0.024, which remains significant at the 1% level.

Beyond statistical significance, the magnitude of the estimated effect is also economically relevant. Relative to the sample mean efficiency score of 0.890, the coefficient of −0.024 is equivalent to approximately 2.7% of the sample mean efficiency score. Although the magnitude appears moderate, it indicates an economically meaningful negative difference considering that the efficiency score is bounded and reflects the overall performance of healthcare resource utilization at the provincial level.

These results are robust and imply that, in its early stage, the provincial pooling reform did not improve medical resource allocation efficiency. Instead, it shows a negative association during the study period.

### 3.4. Robustness Tests

To ensure the robustness of the results, a series of robustness checks were conducted:

(1) Parallel Trends Test.

**Figure 4:**
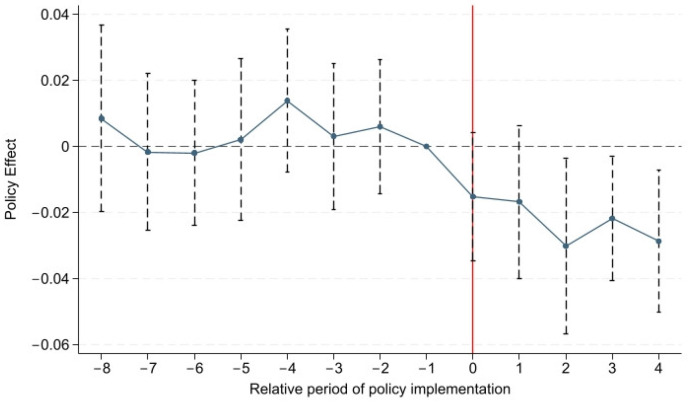
Event-study estimates of the dynamic effects of URRBMI provincial pooling on medical resource allocation efficiency.

To further assess the validity of the staggered DID design, we conduct an event-study analysis to examine the parallel trends assumption. As shown in Figure 4, the estimated coefficients before the implementation of provincial pooling are statistically insignificant, indicating no evidence of differential pre-treatment trends between treated and untreated provinces. The post-treatment effects are generally consistent with the baseline DID estimates, supporting the robustness of the identified policy effects.

(2) Placebo Test

**Figure 5:**
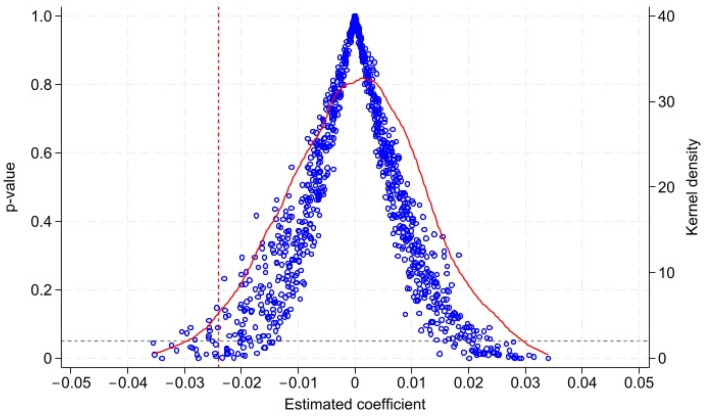
Placebo test results based on 1000 random policy assignments. NOTE: The vertical dashed line indicates the estimated coefficient from the baseline DID model, and the horizontal dashed line represents the 5% significance threshold. The red curve represents the kernel density distribution of placebo estimates.

To further examine whether the estimated effect could be driven by random factors, we conducted a placebo test by randomly assigning treatment status and policy implementation timing. The procedure was repeated 1000 times, and the distribution of placebo estimates is presented in Figure 5. The placebo coefficients are centered around zero, while the benchmark estimate is located in the lower tail of the simulated distribution. These results indicate that the observed negative effect of provincial pooling is unlikely to be driven by random chance, further supporting the robustness of the baseline findings.

(3) Bacon Decomposition

**Figure 6:**
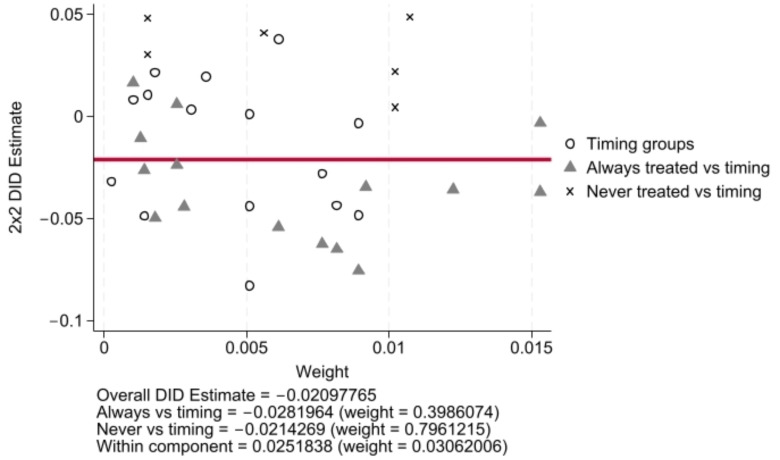
Goodman–Bacon decomposition of the staggered difference-in-differences estimator. NOTE: The red horizontal line indicates the overall DID estimate from the two-way fixed-effects model.

Because staggered TWFE estimates may be affected by heterogeneous treatment timing, we apply the Goodman–Bacon decomposition to examine the composition of the overall TWFE estimator and the contribution of different comparison components [36]. As shown in Figure 6, the overall decomposition estimate is negative (−0.02098), which is consistent with the baseline DID estimate. The decomposition indicates that the overall effect is mainly composed of comparisons across different treatment timing groups, while the contribution of comparisons involving already-treated groups is relatively limited. The signs of the major comparison components are broadly consistent with the overall estimate, suggesting that the baseline findings are not driven by a specific comparison component within the staggered DID framework.

(4) Heterogeneous Treatment Effect Robustness Based on CSDID

To address potential bias arising from heterogeneous treatment effects in staggered DID settings, we further employed the multi-period doubly robust DID estimator proposed by Callaway and Sant’Anna (2021) as a robustness check. This method estimates treatment effects separately for different treatment cohorts and time periods and then aggregates them using appropriate weighting schemes, thereby effectively mitigating the bias that may arise in traditional two-way fixed effects (TWFE) estimators when treatment effects are heterogeneous across groups and over time [37]. As reported in Table 6, the estimated treatment effects remain consistently negative across different aggregation approaches and are broadly consistent with the baseline DID results. These findings suggest that the estimated adverse effect of provincial pooling is robust to alternative DID estimators and is unlikely to be driven by heterogeneous treatment effects.

In addition, several supplementary robustness checks were conducted to further examine the reliability of the baseline findings. Specifically, we controlled for other concurrent policy reforms implemented during the study period, included lagged efficiency as an additional control to account for the persistence of medical resource allocation efficiency, and re-estimated the model using province-clustered standard errors to assess the robustness of statistical inference. The detailed procedures and results of these robustness checks are reported in the Supplementary Materials.

### 3.5. Heterogeneity Analysis

To examine the regional heterogeneity in the policy effects of provincial pooling of the resident basic medical insurance fund, this study divides the full sample into three subsamples (eastern, central, and western regions) following China’s official classification of the three major economic belts. Separate regressions were then estimated for each regional subsample, and the results are reported in Table 7.

The results reveal substantial regional variation in the effects of provincial pooling. The estimated coefficient is significantly negative in western provinces, indicating that provincial pooling reduced medical resource allocation efficiency by approximately 0.055 points. Whereas the effect in eastern provinces is negative but statistically insignificant. No regional estimate is available for central provinces because none implemented provincial pooling during the study period. These findings suggest that the impact of pooling reform is closely related to regional governance capacity and institutional conditions. Compared with eastern provinces, western regions generally face greater challenges in fiscal capacity, healthcare resource distribution, and administrative coordination, potentially making them more vulnerable to the adjustment costs associated with the reform.

To explore heterogeneity in the policy effects of URRBMI provincial pooling across different financing and administrative arrangements, this study further conducts heterogeneity analyses along two institutional dimensions: the fund pooling model and the administrative management model. The results are reported in Table 8.

The estimates are negative under both fund pooling models, but only the risk-adjustment estimate is marginally significant; the revenue–expenditure estimate is smaller and statistically insignificant These findings suggest that differences in fund pooling models may influence both the magnitude and statistical detectability of short-term efficiency changes following provincial pooling reform.

Further heterogeneity analysis by administrative management model indicates that the negative impact of provincial pooling on medical resource allocation efficiency remains statistically significant across different management regimes. The efficiency-reducing effect is more pronounced under the tiered management model, while the negative impact is relatively smaller under vertical management. This pattern suggests that although variations in administrative management structures do not change the direction of the policy effect, they may moderate the extent of efficiency losses associated with the provincial pooling reform.

## 4. Discussion

### 4.1. Main Findings

This study provides empirical evidence that, contrary to policy expectations, provincial pooling of the Urban–Rural Resident Basic Medical Insurance fund is associated with a statistically significant decline in the technical efficiency of medical resource allocation in the short to medium term. A series of robustness checks yields results consistent with the baseline estimate.

The analysis also reveals substantial regional heterogeneity among provinces implementing provincial pooling. The negative effect of provincial pooling is concentrated in western provinces, the eastern estimate is statistically insignificant, and the central effect is not estimable because no central province adopted the reform. These findings suggest that the impact of provincial pooling varies across regional contexts among adopting provinces rather than producing uniform effects nationwide. Institutional heterogeneity analysis further shows that the estimated effects remain negative across fund pooling and administrative management models, but statistical significance differs across arrangements: the revenue–expenditure pooling estimate is not statistically significant, whereas the risk adjustment pooling estimate remains marginally significant.

From a broader perspective, raising the level of risk pooling is widely regarded as a key strategy for integrating fragmented health insurance systems and improving risk sharing. China’s move toward provincial pooling is therefore consistent with international reform trends. However, unlike reforms in more centralized systems, China’s approach has been implemented gradually within a highly decentralized fiscal and administrative structure, where multiple pooling arrangements coexist. This institutional context implies that expanding the risk pool alone is insufficient to improve efficiency and that reform outcomes depend critically on complementary governance arrangements.

Importantly, these findings should be interpreted as reflecting the short- to medium-term consequences of provincial pooling rather than its long-term equilibrium effects. Provincial pooling represents a major institutional restructuring process involving changes in fund management, intergovernmental fiscal relations, and administrative coordination mechanisms. As such, part of the observed decline in efficiency may be attributable to transitional adjustment costs arising during the implementation phase. The long-term effects of the reform may differ as institutional arrangements mature, governance capacity improves, and complementary reforms are progressively integrated.

### 4.2. Mechanisms

The baseline results indicate that URRBMI provincial pooling was associated with a short-term decline in medical resource allocation efficiency. Although the available provincial-level data do not allow for direct empirical identification of specific mechanisms, several institutional perspectives may help explain this unexpected negative effect.

First, the negative effect observed in the baseline estimation may partly reflect the adjustment costs associated with restructuring fiscal authority and administrative responsibilities within the health insurance system. Fiscal federalism theory suggests that the allocation of fiscal functions across different levels of government involves a trade-off between risk sharing and local responsiveness. Lower-level governance structures allow local governments to capitalize on their informational advantages and adjust resource allocation according to local healthcare needs, demographic characteristics, and fund operation conditions [38,39]. By contrast, higher-level pooling enhances risk-sharing capacity and facilitates interregional redistribution. Although provincial pooling expands the scope of risk sharing and strengthens the financial sustainability of the insurance fund, it also requires deeper institutional integration across jurisdictions in areas such as budget management, fund adjustment, and regulatory oversight. Given substantial regional differences in economic development, healthcare resource endowments, and governance capacity, a more centralized management structure may weaken the informational advantages of local governments while generating additional coordination costs and information frictions [40]. During the transition, new rules and coordination requirements may temporarily reduce the flexibility and responsiveness of resource allocation.

Second, the upward transfer of fund management responsibilities may reshape the incentive structures faced by local governments and healthcare providers. Within the operation of the medical insurance system, central and provincial healthcare security authorities are responsible for institutional design and fund management, local governments are responsible for policy implementation and supervision, and healthcare providers deliver medical services. Together, these actors form a hierarchical governance chain characterized by delegated authority and implementation responsibilities. Such an arrangement constitutes a typical multi-level principal–agent relationship, in which higher-level authorities act as principals and local governments and healthcare providers function as agents responsible for achieving policy objectives [41]. Principal–agent theory suggests that as management chains become longer and information is dispersed across multiple organizational levels, principals face increasing difficulties in monitoring agents’ behavior, giving rise to information asymmetry and incentive misalignment [42,43]. As decision-making authority becomes increasingly centralized at the provincial level, the managerial distance between fund administrators and healthcare providers expands, making it more difficult for higher-level authorities to fully observe local healthcare activities and provider behavior. At the same time, the upward transfer of fiscal responsibility may weaken local governments’ incentives to actively supervise healthcare providers and contain medical expenditures. Under these conditions, healthcare institutions may face altered incentives regarding service provision and cost control. Such behaviors reflect agents’ incentives to maximize their own interests when oversight mechanisms are weakened. Consequently, provincial pooling may inadvertently amplify moral hazard problems while enhancing risk-sharing capacity, particularly when governance and monitoring arrangements do not evolve accordingly. When supervisory arrangements and incentive mechanisms fail to evolve alongside the reform, opportunistic behavior arising from information asymmetry may offset some benefits of broader pooling and may be one possible explanation for the negative association observed in the data.

Third, the observed decline in efficiency may also be related to changes in local cost-control incentives after the redistribution of fiscal responsibility. If the redistribution of fiscal responsibility is not accompanied by corresponding accountability mechanisms, provincial pooling may also weaken local governments’ incentives to control costs and improve efficiency. In the operation of medical insurance funds, fiscal responsibility is closely linked to management incentives, as actors bearing financial risks typically have stronger motivations to contain costs and optimize resource allocation. Under lower-level pooling arrangements, local governments directly bear the consequences of fund deficits and rapid expenditure growth, which creates strong incentives for cost control and efficient management. Following provincial pooling, however, part of the financial risk is transferred to the provincial level, thereby reducing the direct fiscal constraints faced by local governments. This shift in the relationship between risk-bearing and accountability may foster expectations of financial support from higher-level authorities. When local governments anticipate that a greater share of fiscal pressure will be absorbed at the provincial level, their incentives to contain expenditure growth and improve management efficiency may weaken accordingly. This phenomenon resembles the “soft budget constraint” commonly discussed in the public finance literature, whereby organizations expecting external financial support tend to exhibit weaker incentives for cost containment and efficiency enhancement [44]. In the absence of synchronized reforms in provider payment systems, medical service pricing mechanisms, and regulatory frameworks, the potential benefits of broader risk pooling may be offset by weakened cost-control incentives, potentially contributing to lower observed efficiency.

The heterogeneity analysis further indicates that the negative effects of provincial pooling are concentrated primarily in western China, whereas no statistically significant effects are observed in eastern provinces. The regional pattern may reflect differences in fiscal, healthcare, and administrative capacity. Western provinces may face greater transitional coordination costs, while provinces with stronger governance systems may be better positioned to manage institutional integration. This interpretation remains tentative because these mechanisms are not directly tested.

### 4.3. Implications for Policy and Practice

The findings of this study suggest that raising the pooling level of the URRBMI fund does not automatically translate into improvements in medical resource allocation efficiency, supporting a phased and institutionally differentiated approach to provincial pooling. International experience likewise suggests that broader risk pooling requires aligned governance, payment, information, and regulatory systems. Thus, risk-sharing gains should not be expected to translate automatically into efficiency gains during institutional transition.

First, provincial pooling should be implemented through regionally differentiated and phased strategies rather than uniform administrative mandates. Entry conditions and transition pathways may need to consider regional differences in administrative capacity, fund management capability, and institutional readiness. Particular attention should be given to western provinces, where efficiency losses appear more pronounced in the short term. Strengthening governance capacity and supporting systems may help facilitate the further development of pooling arrangements.

Second, incentive-compatible mechanisms should be incorporated into pooling reform to mitigate potential moral hazard and improve accountability. Future reforms may consider strengthening the linkage between fund management arrangements and performance-related objectives. Meanwhile, complementary reforms, including provider payment and budget management reforms, may also warrant consideration to ensure policy coherence.

Third, governance capacity and policy coordination should be strengthened to support the sustainable operation of provincial pooling. Investments in integrated information systems, standardized data sharing, and regulatory capacity may contribute to improving fund management and reducing information asymmetry. In addition, continuous monitoring and evaluation should be embedded into the reform process, allowing policymakers to identify implementation challenges and adjust policies accordingly.

Overall, the effectiveness of provincial pooling depends not only on expanding the risk pool but also on the alignment of institutional capacity, incentive structures, and complementary health system reforms.

### 4.4. Strengths and Limitations

This study contributes to the literature by providing macro-level evidence on the efficiency effects of increasing the pooling level of medical insurance funds. Using provincial panel data from 2010 to 2022, it offers a relatively comprehensive assessment of how institutional changes in health insurance financing affect resource allocation outcomes. Methodologically, the combination of a super-efficiency slack-based measure model with a staggered DID design allows for a more nuanced and robust evaluation of efficiency and causal effects.

However, several limitations should be acknowledged. First, the use of provincial-level aggregate data limits the ability to directly observe micro-level behavioral responses and underlying mechanisms. In addition, the limited number of provincial units may reduce the statistical power of subgroup analyses, and the heterogeneous effects should therefore be interpreted with caution. Second, this study does not incorporate patient-level clinical information, such as comorbidities, disease severity, treatment patterns, or individual health outcomes. Consequently, the analysis captures provincial-level technical efficiency in medical resource utilization rather than patient-level clinical outcomes or treatment efficiency. In addition, although the SBM model incorporates multiple input and output indicators, efficiency estimates based on aggregate statistical data may not fully reflect variations in healthcare quality, provider-level heterogeneity, or other dimensions of healthcare performance. Third, this study focuses on short- to medium-term effects, and the long-term impact of pooling reform may evolve as institutional arrangements mature and complementary reforms are implemented. Fourth, despite the robustness checks performed in this study, potential endogeneity concerns remain. As provincial pooling reforms were not randomly assigned, the estimated effects may still be influenced by unobserved provincial factors.

Future research could incorporate micro-level data, such as hospital- or patient-level information, to better identify behavioral mechanisms and distributional effects. Extending the observation period would also allow for a more comprehensive assessment of the dynamic impact of pooling reform and the role of alternative governance arrangements.

## 5. Conclusions

This study provides empirical evidence on the short- to medium-term effects of provincial pooling of the Urban–Rural Resident Basic Medical Insurance (URRBMI) fund on the technical efficiency of medical resource allocation efficiency in China. Contrary to the expectation that expanding the pooling level would automatically improve efficiency, our findings indicate that provincial pooling was associated with a significant decline in technical efficiency during the study period. Moreover, the heterogeneity analysis shows that the negative estimate was statistically significant primarily in western provinces, while no statistically significant effects were observed in eastern provinces, suggesting that the estimated pattern may vary with regional institutional conditions.

These findings contribute to the understanding of health insurance pooling reforms by highlighting that increasing the pooling level alone automatically produces efficiency gains. Provincial pooling represents a broader institutional transformation involving fiscal responsibility, governance structures, and incentive mechanisms. Without corresponding improvements in administrative coordination, accountability arrangements, and supporting reforms, transitional adjustment costs and governance frictions may offset the potential benefits of enhanced risk sharing.

The findings imply that policymakers should move beyond a uniform expansion of pooling levels and adopt context-sensitive reform strategies. Strengthening governance capacity, aligning incentives, improving information systems, and coordinating pooling reform with payment and provider management reforms are essential for realizing the long-term benefits of risk pooling.

## Figures and Tables

**Table 1:** Timeline and institutional design of provincial pooling of URRBMI fund across selected provinces.

Province	Year of Pooling	Fund Pooling Model	Fund Management Model
Shanghai	2016	Revenue–Expenditure Pooling	Vertical Management
Beijing	2018	Revenue–Expenditure Pooling	Tiered Management
Tianjin	2010	Revenue–Expenditure Pooling	Vertical Management
Chongqing	2011	Risk Adjustment Pooling	Tiered Management
Xizang	2018	Revenue–Expenditure Pooling	Tiered Management
Hainan	2015	Revenue–Expenditure Pooling	Vertical Management
Ningxia	2015	Risk Adjustment Pooling	Tiered Management
Fujian	2021	Revenue–Expenditure Pooling	Tiered Management
Qinghai	2016	Risk Adjustment Pooling	Tiered Management
Zhejiang	2022	Risk Adjustment Pooling	Tiered Management
Jiangsu	2021	Revenue–Expenditure Pooling	Vertical Management

NOTE: URRBMI refers to Urban–Rural Resident Basic Medical Insurance.

**Table 2:** Input-output indicator system for measuring medical resource allocation efficiency based on the super-efficiency SBM model.

Indicator Type	Indicator Name	Indicator Definition	Unit
Input Indicators	Number of Medical and Health Institutions	Total number of healthcare institutions in the region.	Institutions
	Number of Health Technicians	Total number of licensed physicians, nurses, and other health professionals.	Persons
	Number of Hospital Beds	Total number of fixed hospital beds.	Beds
Desirable Output Indicators	Number of Inpatients	Total number of patients receiving inpatient care.	10,000 persons
	Number of Surgical Procedures	Total number of surgical operations performed.	10,000 procedures
	Number of Medical Visits	Total number of outpatient and emergency visits.	100 million visits
	Bed Utilization Rate	Ratio of actual bed-days used to available bed-days.	%
Undesirable Output Indicators	Emergency Mortality Rate	Share of deaths among emergency department visits.	%
	Observation Room Mortality Rate	Share of deaths among patients in observation rooms.	%

**Table 3:** Descriptive Statistics of SBM Input, Desirable Output, and Undesirable Output Indicators.

Indicator Type	Variables	Obs	Mean	SD	Min	Max
Input Indicators	Number of Medical and Health Institutions	403	31,867.99	22,340.18	4129	90,194
	Number of Health Technicians	403	278,292.8	187,121.6	9300	916,300
	Number of Hospital Beds	403	239,037.2	161,685.6	8400	752,200
Desirable Output Indicators	Number of Inpatients	403	694.006	491.544	14.55	2021.72
	Number of Surgical Procedures	403	172.723	149.308	2.030	1009.56
	Number of Medical Visits	403	2.466	1.934	0.100	8.920
	Bed Utilization Rate	403	81.744	9.215	48.300	99.300
Undesirable Output Indicators	Emergency Mortality Rate	403	0.089	0.072	0.000	0.400
	Observation Room Mortality Rate	403	0.156	0.364	0.000	2.900

**Table 4:** Descriptive Statistics of Variables Used in the Staggered DID Analysis.

Variables	Obs	Mean	SD	Min	Max
the efficiency of medical resource allocation	403	0.890	0.088	0.576	1.000
Per capita GDP	403	55,964	30,428	12,882	189,988
Social consumption level	403	0.391	0.065	0.180	0.610
Share of tertiary industry	403	49.133	9.215	32.460	83.900
Population density	403	454.018	698.352	2.448	3951.476
URRBMI enrollees	403	2006.108	2141.334	15.000	9183.000

**Table 5:** Baseline difference-in-differences estimates of the impact of URRBMI provincial pooling on medical resource allocation efficiency.

Variables	Medical Resource Allocation Efficiency	Medical Resource Allocation Efficiency
DID	−0.021 ***	−0.024 ***
	(0.008)	(0.007)
Per capita GDP		0.000 ***
		(0.000)
Social consumption level		0.151 ***
		(0.039)
Share of tertiary industry value added		0.001 *
		(0.001)
Population density		0.000
		(0.000)
Number of URRBMI enrollees		0.000 ***
		(0.000)
Constant	0.894 ***	0.677 ***
	(0.002)	(0.046)
Observations	403	403
R-squared	0.880	0.897
Province FE	YES	YES
Year FE	YES	YES

NOTE: *** *p* < 0.01, ** *p* < 0.05, * *p* < 0.1. DID refers to differences-in-differences.

**Table 6:** Heterogeneous Treatment Effect Robustness Based on CSDID.

Variables	(1)	(2)	(3)	(4)
	Simple ATT	Dynamic ATT	Calendar ATT	Group ATT
Simple ATT	−0.0377 ***			
	(0.0116)			
Pre_avg		−0.0068		
		(0.0041)		
Post_avg		−0.0407 ***		
		(0.0091)		
CAverge			−0.0373 ***	
			(0.0093)	
GAverage				−0.0291 *
				(0.0149)

NOTE: *** *p* < 0.01, ** *p* < 0.05, * *p* < 0.1. Simple ATT denotes the overall average treatment effect on the treated estimated using the Callaway and Sant’Anna (2021) estimator. Dynamic ATT reports average pre-treatment and post-treatment effects. Calendar ATT aggregates treatment effects across calendar years, while Group ATT aggregates treatment effects across treatment cohorts.

**Table 7:** Heterogeneous effects of URRBMI provincial pooling by region.

Variables	(1)	(2)	(3)
	Eastern Provinces	Central Provinces	Western Province
DID	−0.014	N.A.	−0.055 ***
	(0.010)		(0.013)
Constant	0.274 *	−0.004	0.961 ***
	(0.146)	(0.335)	(0.121)
Observations	143	130	130
R-squared	0.922	0.909	0.924
Control variables	YES	YES	YES
Province FE	YES	YES	YES
Year FE	YES	YES	YES

NOTE: *** *p* < 0.01, ** *p* < 0.05, * *p* < 0.1. Not estimable due to the absence of treated provinces in the central region.

**Table 8:** Heterogeneous effects of URRBMI provincial pooling by Pooling and Management Models.

	(1)	(2)	(3)	(4)
Variables	Vertical Management	Tiered Management	Revenue–Expenditure Pooling	Risk Adjustment Pooling
DID	−0.023 *	−0.036 ***	−0.012	−0.025 *
	(0.011)	(0.013)	(0.011)	(0.013)
Constant	0.530 ***	0.657 ***	0.624 ***	0.631 ***
	(0.159)	(0.123)	(0.144)	(0.125)
Observations	52	91	91	52
R-squared	0.974	0.917	0.945	0.933
Control variables	YES	YES	YES	YES
Province FE	YES	YES	YES	YES
Year FE	YES	YES	YES	YES

NOTE: *** *p* < 0.01, ** *p* < 0.05, * *p* < 0.1. The institutional heterogeneity analysis is restricted to provinces that implemented URRBMI provincial pooling during the sample period.

## Data Availability

Data are available from the corresponding author upon reasonable request.

## Supplementary Materials

The following supporting information can be downloaded at: https://www.mdpi.com/article/10.3390/healthcare14152368/s1, Figure S1: Event-study estimates of the dynamic effects of URRBMI provincial pooling on medical resource allocation efficiency; Figure S2: Placebo test results based on 1000 random policy assignments; Figure S3: Goodman–Bacon decomposition of the staggered difference-in-differences estimator; Table S1: Heterogeneous Treatment Effect Robustness Based on CSDID; Table S2: Robustness checks controlling for concurrent health system reforms; Table S3: Robustness check controlling for lagged efficiency; Table S4: Robustness Check Using Province-Clustered Standard Errors.

## Abbreviations

The following abbreviations are used in this manuscript:

URRBMI	Urban–Rural Resident Basic Medical Insurance
NHSA	National Healthcare Security Administration
SBM	Slack-Based Measure
DEA	Data Envelopment Analysis
DID	Difference-in-Differences
