# Supplementary material for: Does Provincial Pooling of the Resident Medical Insurance Fund Improve the Efficiency of Medical Resource Allocation of China?
Source: Healthcare (Basel). 2026 Aug 3;14(15):2368. doi: 10.3390/healthcare14152368 (PMC13465428; doi:10.3390/healthcare14152368)
Supplement: Supplementary file 1 [file healthcare-14-02368-s001.zip › healthcare-4429513-supplementary.pdf]

(1) Parallel Trends Test

To examine the validity of the staggered DID design, this study conducts an event-study analysis to test the parallel trends assumption before the implementation of URRBMI provincial pooling. The period immediately before policy implementation is selected as the reference period, and the dynamic effects of provincial pooling are estimated for periods before and after the reform.

As shown in **Figure S1**, the estimated coefficients for the pre-treatment periods are close to zero, and their 95% confidence intervals all include zero, indicating that there were no statistically significant differences in the trends of medical resource allocation efficiency between provinces that adopted provincial pooling and those that had not yet implemented the reform before policy implementation. These results provide sup-port for the validity of the parallel trends assumption required for the DID estimation.

After the implementation of provincial pooling, the estimated coefficients become negative and remain below zero in subsequent periods, suggesting that the reform was associated with a decline in medical resource allocation efficiency over time. The dy-namic effects are generally consistent with the baseline DID results, further confirming the robustness of the estimated policy effects.presents the estimated dynamic effects of the URRBMI provincial pooling reform on medical resource allocation efficiency. The coefficients in the pre-policy period fluctuate around zero and are statistically insignificant, indicating that the parallel trends assumption is satisfied. This evidence supports the validity of the identification strategy. Following policy implementation, the coefficients become significantly negative and remain so over time, demonstrating that the provincial pooling reform has a substantial and persistent adverse impact on medical resource allocation efficiency.

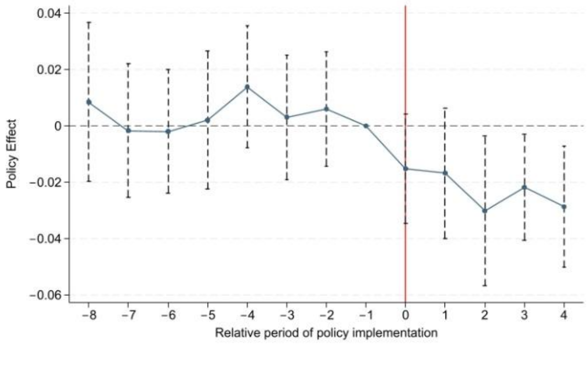

Figure S1. Event-study estimates of the dynamic effects of URRBMI provincial pooling on medical resource allocation efficiency

(2) Placebo Test

To further verify the reliability of the benchmark findings and rule out the possibility that the estimated effect was driven by random factors, a placebo test was conducted following the approach commonly adopted in the DID literature. During the sample period, 12 provinces implemented provincial pooling of the Urban–Rural Resident Basic Medical Insurance (URRBMI) fund. Accordingly, in each simulation, 12 provinces were randomly selected (without replacement) from all provinces to form a hypothetical treatment group, matching the number of actual policy adopters. Meanwhile, pseudo-policy implementation years were randomly assigned, and the baseline specification was re-estimated using the resulting placebo treatment indicator.

This procedure was repeated 1000 times, generating 1000 placebo coefficients and corresponding p-values. Figure S2 presents the distribution of the placebo estimates. The placebo coefficients are approximately centered around zero, indicating that randomly assigning treatment status and policy timing does not systematically generate treatment effects. By contrast, the benchmark estimate (−0.024) is located in the left tail of the simulated distribution. Only 26 out of 1,000 simulations (2.6%) produced coefficients more negative than the benchmark estimate. These results suggest that the probability of obtaining the benchmark finding purely by chance is low, providing further support for the validity and robustness of the baseline results.

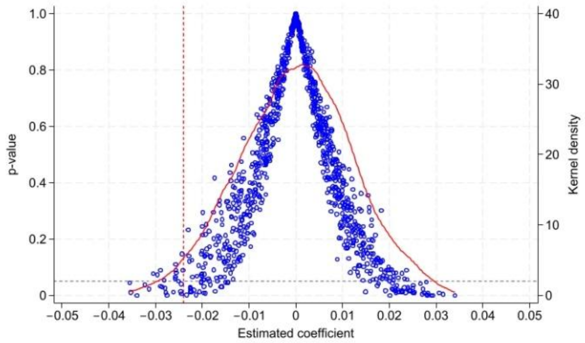

Figure S2. Placebo test results based on 1000 random policy assignments

(3) Bacon Decomposition

To assess potential bias arising from heterogeneous treatment effects in the staggered DID model, we employ the Bacon decomposition to diagnose the estimator. The results are presented in Figure S3. The decomposition shows that the weights of the 2×2 DID components are small and concentrated, with no negative or anomalous weights observed. Most of the 2×2 estimates cluster around zero and align in direction with the overall estimate,

indicating no evidence of bias arising from inappropriate comparisons.

The overall DID estimate (–0.0209) is largely consistent with the component-specific estimates, suggesting that heterogeneous treatment effects exert only a limited influence on the two-way fixed effects estimator. These findings support the robustness of the estimated policy effect.

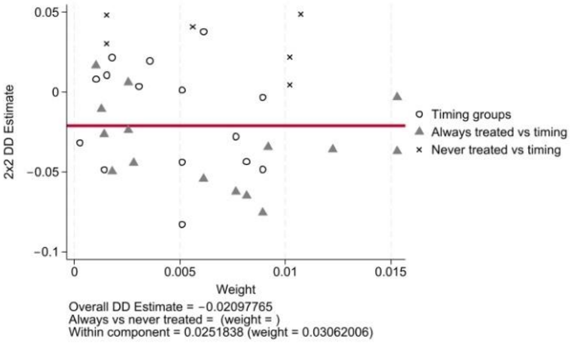

Figure S3. Goodman–Bacon decomposition of the staggered difference-in-differences estimator

(4) Heterogeneous Treatment Effect Robustness Based on CSDID

To further address potential bias arising from heterogeneous treatment effects in staggered DID settings, we employed the multi-period doubly robust estimator proposed by Callaway and Sant'Anna (2021) as an additional robustness check.

The results are reported in Table S1. Across all aggregation approaches, including the simple average treatment effect, dynamic treatment effects, calendar-time average treatment effects, and group-specific average treatment effects, the estimated effects remain consistently negative and broadly aligned with the baseline findings. Overall, these results provide further evidence that the adverse effect of provincial pooling on the efficiency of medical resource allocation is robust and is unlikely to be driven by heterogeneous treatment effects in the staggered DID framework.

Table S1. Heterogeneous Treatment Effect Robustness Based on CSDID

VARIABLES	(1)<br>Simple ATT	(2)<br>Dynamic ATT	(3)<br>Calendar ATT	(4)<br>Group ATT
Simple ATT	-0.0377***			
	(0.0116)			
Pre_avg		-0.0068		
		(0.0041)		
Post_avg		-0.0407***		
		(0.0091)		
CAverge			-0.0373***	

	(0.0093)	
GAverage		-0.0291*
		(0.0149)

NOTE: *** p<0.01, ** p<0.05, * p<0.1 Simple ATT denotes the overall average treatment effect on the treated estimated using the Callaway and Sant'Anna (2021) estimator. Dynamic ATT reports average pre-treatment and post-treatment effects. Calendar ATT aggregates treatment effects across calendar years, while Group ATT aggregates treatment effects across treatment cohorts.

(4) Controlling for Other Concurrent Policies

To isolate the independent effect of the URRBMI provincial pooling reform and eliminate potential confounding from other major health reform policies implemented during the same period, we incorporated three categories of important policy variables into the baseline model. The estimation results are reported in Table S2. After controlling for these concurrent policies, the negative impact of provincial pooling on medical resource allocation efficiency remains significant and robust, indicating that the observed suppressive effect is not driven by competing reforms. This finding provides a reliable empirical basis for accurately assessing the policy impact of provincial pooling within China’s medical insurance system.

Table S2. Robustness checks controlling for concurrent health system reforms

	(1)	(2)	(3)
VARIABLES	Provincial Pooling of Urban Employee Insurance	Provincial Comprehensive Healthcare Reform	Regional Medical Centers
DID	-0.024***	-0.023***	-0.024***
	(0.008)	(0.007)	(0.007)
Observations	403	403	403
R-squared	0.897	0.898	0.897
Control variables	YES	YES	YES
Province FE	YES	YES	YES
Year FE	YES	YES	YES

NOTE: *** p<0.01, ** p<0.05, * p<0.1

(4) Controlling for Lagged Efficiency

To further alleviate concerns regarding potential endogeneity and reverse causality, we re-estimated the baseline model by including the one-period lagged value of medical resource allocation efficiency. This specification accounts for the persistence of efficiency outcomes over time and reduces the possibility that

provinces with pre-existing efficiency problems were more likely to adopt the provincial pooling reform.

The estimation results are reported in Table S3. The coefficient of the provincial pooling reform variable remains negative and statistically significant after controlling for lagged efficiency, indicating that the baseline findings are not driven by pre-existing differences in efficiency levels across provinces. These results provide additional support for the robustness of the estimated policy effect and strengthen the causal interpretation of the main findings.

Table S3. Robustness check controlling for lagged efficiency

VARIABLES	(1)<br>Efficiency	(2)<br>Efficiency
DID	-0.0178**	
	(0.007)	
L.DID		0.563***
		(0.049)
Observations	372	372
R-squared	0.935	0.935
Control variables	YES	YES
Province FE	YES	YES
Year FE	YES	YES

NOTE: *** p<0.01, ** p<0.05, * p<0.1

(5) Robustness Check Using Province-Clustered Standard Errors

To address concerns regarding statistical inference arising from the relatively small number of cross-sectional units, we re-estimated the baseline specification using province-clustered standard errors. Clustering at the provincial level allows for arbitrary serial correlation and heteroskedasticity within provinces over time. The estimated coefficient on DID remains negative and statistically significant, indicating that the main findings are not sensitive to the variance estimation method(see Table S4).

Table S4. Robustness Check Using Province-Clustered Standard Errors.

VARIABLES	(1)<br>Efficiency
DID	-0.024**
	(0.010)
Observations	403
R-squared	0.897
Clusters	31
Control variables	YES
Province FE	YES
Year FE	YES
